# The Role of Culture, Environment, and Religion in the Promotion of Physical Activity Among Arab Israelis

**Published:** 2008-06-15

**Authors:** Kerem Shuval, Eyal Weissblueth, Araida Amira, Mayer Brezis, Zubaida Faridi, Ather Ali, David L Katz

**Affiliations:** Yale-Griffin Prevention Research Center. Dr Shuval is also affiliated with Ohalo College; Ohalo College, Qatzrin, Israel; Ohalo College, Qatzrin, Israel; Center for Quality & Safety, Hadassah-Hebrew University Hospital, Jerusalem, Israel and Physical Activity Promotion Committee, National Council for Health Promotion, Ministry of Health, Jerusalem, Israel; Yale-Griffin Prevention Research Center, Derby, Connecticut; Yale-Griffin Prevention Research Center, Derby, Connecticut; Yale-Griffin Prevention Research Center, Derby, Connecticut

## Abstract

**Introduction:**

Despite low levels of physical activity among Arabs in Israel, interventions designed to increase physical activity in this population have been scarce. To improve our understanding of the cultural, religious, and environmental barriers and enablers to physical activity, we conducted a qualitative study among Arab Israeli college students in Israel.

**Methods:**

A total of 45 students participated in 8 focus groups. Purposeful sampling was used to capture the diverse characteristics of participants. Two researchers analyzed the data independently guided by grounded theory. Peer-debriefing sessions were held to group preliminary categories into larger themes. Generally, consensus between researchers was high, and minor differences were resolved.

**Results:**

Participants recognized the importance of physical activity in chronic disease prevention, yet most were not regularly physically active. This contradiction could be explained by the fact that many participants lived in an extended-family setting that deemphasized the importance of physical activity. Women often found themselves exercising at odd hours so that they would not be noticed by neighbors. Religion, in comparison, was considered a facilitating factor because the scriptures supported physical activity. Furthermore, an urban environment was an enabling factor because it provided exercise facilities, sidewalks, and a socially acceptable venue for activity. Participants felt resources were not allocated by the government to accommodate physical activity.

**Conclusion:**

Increasing Arab Israelis' access to safe and culturally appropriate exercise facilities should become a priority. Thus, policy changes in allocating appropriate funds to promote physical activity must be considered, along with using multiple health promotion strategies.

## Introduction

As is the case with ethnic minority populations in other countries, Arabs in Israel have higher rates of diabetes, obesity, hypertension, and coronary heart disease and lower rates of physical activity than do the majority Jewish population (1). In fact, according to the Israeli National Health Interview Survey (2), Jewish men were 1.6 times more physically active than Arab men (35.2% vs 22.4%, respectively), and Jewish women were 1.9 times more physically active than Arab women (33.2% vs 17.0%, respectively), after adjusting for confounders (e.g., sex, age, education). Leisure-time physical activity remained approximately constant in relation to age among Arab men (24.7% of 21- to 24-year-old men were physically active, compared with 26.7% of 65- to 74-year-old men). Conversely, physical activity levels among Arab women decreased with increasing age (22.8% of 21- to 24-year-old women were active, whereas only 8.5% of 65- to 74-year-old women were active).

In response to the findings from the National Health Status Survey that indicated low rates of physical activity and the extensive evidence on the association between physical activity and health (3,4), Israel's Ministry of Health National Council for Health Promotion established a Physical Activity Promotion Committee (PAPC). PAPC consists of national experts in public health, health policy and economics, medicine, and physical activity and nutrition. PAPC adopted the U.S. Centers for Disease Control and Prevention's recommendations for the type, intensity, and duration of activity required to achieve associated health benefits (5). In 2004, PAPC set an objective, similar to *Healthy People 2010* (6), to increase the proportion of the population that obtains the recommended amount of physical activity from 30% to 60% within 10 years, while stressing the need to focus on minority subpopulations.

To date, however, interventions targeted at promoting physical activity among the Arab Israeli population have been scarce, and no studies of these interventions have been published. Furthermore, to the best of our knowledge, no studies have examined Arab Israelis' perceptions of barriers and enablers to physical activity. To remedy this deficiency and in an initial attempt to gain understanding of the needs for physical activity promotion among this minority population, we conducted focus groups among Arab Israeli physical education students in a predominately Arab (64.5%) college in rural northern Israel. We hypothesized that conducting focus groups with future physical education professionals in the Arab Israeli sector might help program planners develop culturally appropriate exercise interventions.

## Methods

We conducted a qualitative focus group study from November 2005 through March 2006 to explore the cultural, religious, and environmental barriers and enablers to physical activity, as well as participants' exercise behavior. To establish validity (7), participants' physical activity — assessed during focus group sessions — was triangulated with quantitative data (our unpublished data) derived from a college-wide survey that used the International Physical Activity Questionnaire (8). Before beginning the study, approval was obtained from the Ohalo College institutional review board.

Of 128 Arab physical education students at Ohalo College, 45 participated in our focus groups. We used purposeful sampling (9) to select key informants (on the basis of researchers' acquaintance), promote group interaction, and capture the diverse characteristics of participants (i.e., sex, age, living environment, ethnicity/religion, and self-reported income). All students whom we approached agreed to participate in focus groups. Slightly more than half of the participants were female, a minority reported being secular, most were Muslim or Bedouin, and most lived in a rural environment (i.e., in rural villages adjacent to Ohalo College) ( Table 1). Mean age of participants was 21.9 years (range 18.0–31.0 years).

Focus groups were held until no new themes emerged (9). We conducted 8 focus groups of 4 to 7 participants each, 90 minutes per session. At least one key informant (previously targeted) was included in each focus group. Two facilitators, an experienced researcher and a bilingual/bicultural moderator, ran the focus groups jointly. The moderator explained the aim of the focus groups and had participants complete a brief demographic questionnaire. A predetermined set of questions and probes was used throughout the sessions (Appendix). These questions were developed and refined with input and insight from 3 focus groups (with 19 Arab Israeli college students) that were conducted before the beginning of the study; the students who helped develop the questions did not participate in the study.

Focus groups were recorded and transcribed verbatim within 72 hours. Researchers reviewed the transcripts to reflect on each session before conducting the next, thereby enabling newly identified concepts to be examined in subsequent sessions. To bolster the trustworthiness of the analysis, two researchers (KS, EW) analyzed the data independently, guided by grounded theory (10). Transcripts were coded and sorted into preliminary categories. Peer-debriefing sessions were held to group preliminary categories into larger themes. Generally, consensus between researchers was high, and minor differences were resolved between them. All researchers took part in the interpretation of the data in relation to the original objectives and emerging themes.

## Results

Participants perceived physical activity to be an integral part of health promotion. They were well aware of PAPC recommendations regarding the need for 30 or more minutes of moderate-intensity physical activity on most days of the week and the associated health benefits. Moreover, physical activity was believed to prevent the onset of chronic diseases, reverse diabetes and hypertension, and improve emotional well-being. Still, many participants admitted that they did not meet PAPC recommendations for activity (Table 2).

All participants emphasized the role social norms play in health behavior change in the Arab population in Israel. Some students noted that participating in culturally acceptable team sports (e.g., soccer) was an appropriate way to stay physically active. Most participants who had lived in large cities, away from their extended families, perceived that an urban living environment helped them maintain a physically active lifestyle. Others suggested that living in an urban environment with Western influences offered more opportunities for exercise but also more temptation to eat in restaurants and avoid exercise (Table 2).

For some students, religious belief was perceived as a facilitator to physical activity. Many Muslim students referred to scriptures from the Quran as promoting a physically active lifestyle. On the other hand, the concept of religious belief and fatalism emerged consistently throughout focus group sessions, particularly from religious and ultrareligious students, who felt that adopting a healthier lifestyle was futile since God determines life expectancy (Table 2).

Both male and female participants believed men were more physically active than women because of the barriers women encounter when attempting to be active. Both female and male participants perceived that women were more inclined to conform to conservative social norms. Furthermore, students added that women were not permitted to exercise without being accompanied by a male companion (husband, father, or brother). Participants did, however, report a recent trend gaining acceptability in the Arab Israeli society in which small groups of women can be seen power-walking in many villages. Other participants suggested that the best way to enable women to be physically active is to designate exercise facilities for women only (Table 2).

Almost all participants believed Jews were more active than Arabs, not only because of cultural limitations in Arab society but also from perceived governmental discrimination. A few participants believed the lack of facilities was due to mismanagement of funds disbursed to their local municipalities (Table 2).

Participants had many suggestions for increasing physical activity in the Arab population. They suggested increasing awareness of the health benefits of exercise within a culturally appropriate context and pointed out the population's susceptibility to chronic diseases. They felt that that type of health promotion intervention could decrease health disparities between the Arab and Jewish population. Participants observed that changing the attitudes and perceptions of family members would help. Finally, participants suggested that by enhancing individual self-efficacy and addressing personal impediments, some cultural barriers could be overcome (Table 2).

## Discussion

To the best of our knowledge, this is the first qualitative study to examine facilitators and barriers to physical activity in the Arab population either in Israel or the Middle East. A number of cross-sectional studies have examined levels of adherence to physical activity in Arab or Muslim populations (1,11,12), but cultural and environmental impediments to physical activity have not been investigated (13). We attempt to bridge this gap by illuminating cultural, environmental, and religious facilitators and barriers to physical activity among Arab Israelis.

Although participants recognized the importance of physical activity in health promotion and chronic disease prevention, most admitted to being not regularly active, which is consistent with findings in a multiethnic sample of older adults (14). The contradiction between awareness of the importance of physical activity and lack of exercise could be explained by the role social norms play as a barrier to physical activity. Intracommunal consensus influences the minority to conform to norms of the majority, particularly when living in an extended-family structure (15). Thus, living in an extended-family setting, which deemphasizes the importance of physical activity, prevented participants from leading a physically active lifestyle. The local community had been on occasion verbally disruptive or abusive of individuals or groups attempting to be physically active. Women, in particular, had to abide by cultural standards and often found themselves exercising in adjacent Jewish towns or at odd hours so that they would not be noticed by neighbors. A social environment conducive to physical activity (e.g., seeing people being physically active in your neighborhood) increases the likelihood of achieving recommended levels of physical activity (16).

Participants perceived religion to be a facilitating factor because the Muslim scriptures justified physical activity. However, quantitative data from a parallel survey revealed no significant difference in physical activity levels between religious and nonreligious Arab students. Furthermore, some religious participants expressed fatalistic views of health, which impede health-promoting behaviors by reducing self-efficacy and increasing external locus of control (17). Fatalism has also been found to act as a barrier to preventive health behavior among other minority populations (e.g., African Americans) and other cultures worldwide (18-21).

The Social Ecological Model (22-24), which acknowledges many factors that influence health behavior, could be used to examine the results of our study. Interpersonal factors, such as social environment and level of social support, had a greater effect on the behavior of study participants than did intrapersonal factors (e.g., attitude, self-efficacy). Female participants, for instance, reported that group camaraderie, rather than self-efficacy, encouraged them to become or stay physically active. Additionally, the Social Ecological Model's emphasis on environment and policy as facilitators of physical activity is consistent with the findings of the study. An urban environment was an enabling factor by providing facilities, sidewalks, and a socially acceptable venue for exercise. In contrast, the rural environment was primarily regarded as an impediment, not only because of the need to conform to social norms but also because appropriate facilities were lacking. Participants felt resources were not allocated by the government or local municipality to accommodate physical activity. Providing access to safe and culturally appropriate exercise facilities has been found to promote physical activity (25,26).

To promote physical activity in the Arab Israeli population, program planners should consider using multiple health promotion strategies, such as social marketing and personal feedback (27). Increasing the population's awareness, along with supporting positive physical activity trends (e.g., walking groups in Arab communities) might lead to a change in social norms, which, in turn, might encourage behavioral change. Moreover, environmental factors must be considered when designing physical activity promotion programs, including culturally appropriate facilities (e.g., separate facilities for women, with female instructors). A community-based approach involving religious leaders might facilitate change, and policy changes in allocating appropriate funds to promote physical activity of the minority population in Israel must be considered as well.

As is the case with other qualitative studies, the primary limitation of this study is the inability to generalize its findings (28). However, focus group participants were demographically similar to the general Arab Israeli population in several ways. For example, 49% of focus group participants were female compared with 53% in the general Arab population. Other comparable variables included living environment (27% urban in focus group participants vs 21% in the general Arab population); physical activity levels of female participants (21% in participants vs 23% in 21- to 24-year-old Arab women); and physical activity levels of male participants (33% vs 25% in 21- to 24-year-old Arab men). The study sample was, however, different from the general Arab population in several ways. Christians were overrepresented in the study sample, and Muslims were underrepresented. Additionally, this sample of future health professionals might be more educated and cognizant of physical activity guidelines and health promotion strategies than the general Arab Israeli population, though this supposition has not been substantiated in national surveys.

## Figures and Tables

**Table 1:** Demographics, General Characteristics, and Physical Activity Level of Arab Israeli Focus Group Participants (N = 45), Northern Israel, 2005–2006

Characteristic	No. (%)
**Sex**	
Male	21 (46.7)
Female	24 (53.3)
**Living environment**	
Urban	12 (26.7)
Rural	33 (73.3)
**Religious practice**	
Secular	9 (20.0)
Observant	21 (46.7)
Religious	8 (17.8)
Ultrareligious	7 (15.6)
**Ethnicity/religion**	
Muslim	17 (37.8)
Bedouin	12 (26.7)
Druz	5 (11.1)
Christian	11 (24.4)
**Household income**	
Low	10 (22.2)
Middle class	23 (51.1)
Upper-middle class	10 (22.2)
High	2 (4.4)
**Meet PAPC guidelines for physical activity^a^**	12 (26.7)
Men who meet guidelines (n = 21)	7 (33.3)
Women who meet guidelines (n = 24)	5 (20.8)

a Physical Activity Promotion Committee (PAPC) guidelines are consistent with those of the U.S. Centers for Disease Control and Prevention: ≥3 days/week of vigorous physical activity for ≥20 minutes/day or ≥5 days/week of moderate physical activity for ≥30 minutes/day.

**Table 2:** Themes Observed From Arab Israeli Focus Groups and Supporting Quotations Regarding the Promotion of Physical Activity, Northern Israel, 2005–2006

**Theme**	**Quotations**
Physical activity and health promotion	"I sneak in 10-minute workouts a few times a day. That's just as effective as a continuous workout." "Yeah, sure I know physical activity has enormous benefits. It affects human physiology, prevents cancer. I know all that. But am I physically active according to the recommended level? No way, not even close."
Social norms and living environment	"Physical activity is not valued in our culture. There just isn't enough awareness of its importance and the linkage to improved health. Parents don't encourage their kids to be involved in sports. Academics is much more important." "If I were to power-walk in broad daylight and my friends saw me, I would be their laughingstock." "I used to jog through the marketplace. I changed my route because my friends would say, 'Hey man, come and play cards with us!'" "If you want to be active in my village then soccer is the way to go. But try walking recreationally, and you'll be ridiculed." "I live in a big city in an Arab neighborhood. Not only are there no real social conformities constraining me, but I have a gym next to my house, and I go to the beach a few times a week and walk along the seashore." "I really feel the Western influence on my lifestyle since we moved here from our village. On the one hand there are a lot of exercise facilities available and plenty of sidewalks to use, but on the other hand I find myself eating out more and exercising less."
Religion, clergy, and fatalism	"The Quran encourages physical activity. Swimming and horseback riding are specifically mentioned in the scriptures." "Physical activity is extremely important, but it doesn't affect one's life expectancy. Only God can determine when it's time to die and when it's time to live." "It's in the hands of Allah. You can be as physically active as you want, eat a balanced diet, and the next day you'll be run over by a truck while jogging. So what's the point?"
Gender and physical activity	"Women are expected to cook, stay at home, clean, and take care of their husband. By the end of the day we're just too tired to do anything." "Women are required to wear traditional clothes when exercising; it isn't a religious issue, but rather a matter of tradition." "A guy can go out at 12 at night and go running, but girls just can't." "Girls will be ostracized if they exercise unaccompanied. I wake up sometimes at 5 am and go for a walk, so no one can see me." "I've been walking 3 times a week with my girlfriends. At first we were made fun of, but then they stopped laughing. We're really committed." "Many more exercise facilities serve men, whereas a rare few set aside hours for women only."
Discrimination and lack of facilities	"[Arab] townships and villages are receiving less funding for sports programs than our Jewish neighbors." "There're just no facilities in my village of 10,000 people. We don't even have a gym or swimming pool. No organized activities exist for children and youth, let alone the adult population." "Our local municipality doesn't understand how important exercise is for the population. They invest most of their budget in erecting a new building for officials but won't invest in sports facilities or programs; that's too bad."
Strategies for increasing physical activity	"If we let them [Arab population] know, maybe through mass media, that exercise can decrease the need for medication, it might have a serious impact on their health and behavior." "Culturally acceptable activities must be offered. We can't promote soccer for women; it just won't work. But offering exercises classes for women only, using female instructors, would definitely work." "Intervention programs must specifically be aimed at increasing the awareness of individual families and the extended family." "I suggest increasing people's confidence to change their sedentary behavior. Let's empower them to overcome personal and cultural issues."

## Appendix. Questions and Probes for Focus Group Sessions of Arab Israeli Physical Education Students (N = 45), Northern Israel, 2005–2006

**1. How do you define physical activity?**

1.1 How does physical activity affect (if at all) your health?

1.2 In your opinion, are you sufficiently physically active? Please elaborate and describe your physical activity routine on a typical week.

**2. What are the main barriers (if at all) or facilitators (if at all) that affect your participation in regular physical activity?**

2.1 How does the physical environment (i.e., traffic, lighting, sidewalks, safety, facilities) impact (if at all) your physical activity routine?

2.2 How does your city, community, or neighborhood affect (if at all) your physical activity routine?

2.3 How does your religious belief or practice influence (if at all) your physical activity behavior? What impact (if at all) do religious clergy have on your behavior?

2.4 What personal factors (e.g., support from friends or family, self-confidence, time, finances) affect your activity the most? Please elaborate.

**3. Which strategies should be employed, in your opinion, to increase your physical activity levels as an individual and within your community?**

**4. Is there anything else you would like to add?**
